# Surgical Management of Thick Primary Cutaneous Melanoma in the US

**DOI:** 10.1002/cam4.70578

**Published:** 2025-02-20

**Authors:** Arthur W. Cowman, Kristel Lourdault, Douglas Hanes, Jessica Weiss, Sean Nassoiy, Melanie Goldfarb, Richard Essner

**Affiliations:** ^1^ Saint John's Cancer Institute Santa Monica California USA; ^2^ Providence Research Network Portland Oregon USA

**Keywords:** NCDB, sentinel lymph node biopsy, surgical management, survival, thick melanoma

## Abstract

**Background:**

There remains significant variability in the surgical management of thick melanoma patients with clinically node‐negative disease. We evaluated factors influencing overall survival (OS) in these patients, focusing on the surgical management of the primary tumor and nodal basin.

**Methods:**

Using the National Cancer Database, we identified 7647 patients diagnosed between 2012 and 2017 with thick melanoma (> 4 mm, T4) and clinically node‐negative disease. 4332 patients had complete data and met all inclusion criteria. These patients were stratified into three groups based on nodal assessment: sentinel lymph node biopsy (SLNB), elective lymphadenectomy (ELND), or no nodal evaluation (NNE). OS was compared using Kaplan–Meier analyses and multivariable Cox proportional hazard regression.

**Results:**

In the cohort, 2851 (65.8%) had a SLNB, 799 (18.4%) had an ELND, and 682 (15.7%) had NNE. OS significantly decreased for each millimeter of increasing Breslow thickness. Ulceration, lymphovascular invasion, and tumor‐positive SLN (+SLN) were associated with worse OS (all *p* < 0.001). The size of surgical margins was not significantly associated with OS. Five‐year OS of patients with SLNB was 67.1% ± 1.2% compared to 57.9% ± 2.3% with ELND and 46.8% ± 2.5% with NNE (*p* < 0.001). Among +SLN patients, a complete lymph node dissection (CLND) was performed in 400 (62.3%) but was not associated with improved OS (*p* = 0.67) when compared to the nodal observation group.

**Conclusion:**

Our results suggest that increasing Breslow thickness and nodal assessment provide important prognostic information regarding OS for thick melanoma patients, which emphasizes the importance of SLNB for staging and confirm the lack of benefit of CLND after +SLN in thick melanoma. The size of surgical margins did not affect OS.

## 1 Introduction

Cutaneous melanoma is an increasing health concern in the United States, with 100,640 new cases and 8290 deaths expected in 2024 [1]. Most patients with a new cutaneous melanoma are diagnosed at early AJCC stage I or II, with less than 5% of patients having thick primary tumors (> 4 mm thickness) [2, 3]. Although the rate of thin (< 1 mm) and intermediate‐thickness (1–4 mm) melanoma has stabilized over the last decade, studies have shown an increase in the incidence of thick melanoma [4]. With melanoma accounting for approximately 75% of skin cancer‐related mortalities, understanding this high‐risk subgroup has become increasingly important as now there is FDA approved adjuvant immunotherapies for high‐risk AJCC stage II melanoma [5, 6, 7]. These findings illustrate the need to investigate ways in which the surgical management of thick melanoma patients can be optimized, regarding the size of the surgical margins of the wide excision and the management of the nodal basin.

Guidelines for patients with primary tumors thicker than 2 mm (T3‐T4) recommend 2 cm surgical margins for wide excision. Reducing the excision margins could reduce the need for skin grafts and morbidity associated with wider margins without changing survival. The ongoing international Melanoma Margins Trial (NCT01285214) is designed to compare 1–2 cm surgical margins for T2b‐T4 patients [8]. In anticipation of the results from the trial, we sought to address excision margins in patients with T4 lesions, specifically.

Aside from patients with poor general health for whom extensive surgery may not be recommended [9], two treatment options can be considered for the management of the regional lymph nodes: sentinel lymph node biopsy (SLNB), and elective lymph node dissection (ELND); the latter has been essentially replaced by SLNB since the first Multicenter Selective Lymphadenectomy Trial (MSLT‐I). The National Comprehensive Cancer Network (NCCN) recommends surgeons discuss and offer a SLNB if there is a greater than 10% chance of a tumor‐positive sentinel lymph node (+SLN), but do not specify treatment for thick melanoma patients and ultimately leaves this decision up to the surgeon [10].

MSLT‐I demonstrated the staging advantages of SLNB over wide excision alone for patients with intermediate‐thickness melanoma and suggested SLNB should be considered for patients with thick melanoma, but the benefits for the latter group should be considered carefully as thick melanoma only represented 15% of the study group [11]. Furthermore, the MSLT‐II and the German Dermatologic Oncology Cooperative Group's (DeCOG‐SLT) randomized clinical trials showed no significant improvement in melanoma‐specific survival or overall survival (OS) for patients who had +SLNs and underwent a subsequent complete lymph node dissection (CLND) compared to those who did not have a CLND following the identification of a + SLN [12, 13]. Both trials had a limited number of patients with thick primary tumors to draw conclusions about CLND. With the limited number of thick melanoma patients included in randomized surgical clinical trials (MSLT‐I and II) [11, 13] the impact of the surgical management of the regional nodal basin on this group of patients is not clearly defined.

Previous studies have tried to assess the surgical management of thick melanoma patients, but all have been hindered by inherent biases. Indeed, either they included a limited number of patients, such as the MSLT‐I, ‐II, and the DeCOG‐SLT [11, 13, 14], or the type of surgery patients received was not clearly defined [15]. This is the case of Song et al.'s study, which used the National Cancer Database (NCDB) cohort before the database had incorporated the type of nodal surgery patients received, incorporating SLNB and ELND into recorded surgical procedures [15].

We elected to use the NCDB, but with a focus on patients diagnosed after 2012, when the NCDB incremented a new code that specifies the type of nodal surgery patients received concurrently with the wide excision of the primary tumor. Thus, our study, based on prospectively collected data from the NCDB, aims to assess which surgical approach provides the best outcome for patients with thick primary melanoma.

## 2 Materials and Methods

### 2.1 Study Design

The NCDB was queried from the years 2012 to 2017 to identify patients ages 18–80 with a diagnosis of thick primary cutaneous melanoma (cT4) who were clinically node‐negative (cN0) and without known metastatic (cM0) disease. The exact Breslow thickness was known for primary tumors > 4 mm and up to 9.8 mm, while thicker primaries were all classified as ≥ 9.8 mm. We used data starting from 2012 because this was when the NCDB added the code RX_SUMM_SCOPE_REG_LN_2012, which specifies the type of nodal surgery patients received concurrently with the wide excision of the primary melanoma tumor.

**FIGURE 1:**
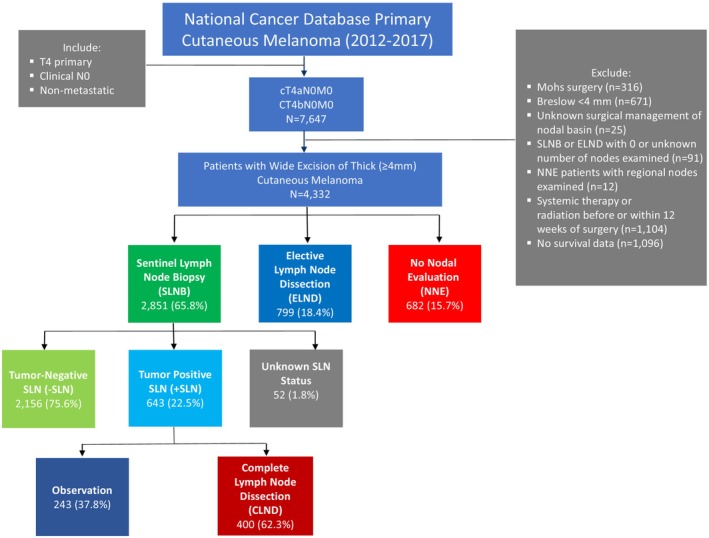
Experimental design and cohort definitions. Seven thousand six hundred forty‐seven patients were identified with thick melanoma (> 4 mm, T4), clinically negative nodes (N0), and non‐metastatic (M0) cancer from the NCDB. Of the 4332 patients included in this study, 2851 underwent a sentinel lymph node biopsy (SLNB), 799 underwent an elective lymph node dissection (ELND), and 682 had no nodal evaluation (NNE).

After identifying 7647 patients who met inclusion criteria, patients were excluded from the analyses if Breslow thickness was verified as less than 4 mm, if they had Mohs surgery for the treatment of their primary, or if OS data was missing. In addition, patients who were miscategorized (e.g., patients who had regional lymph node evaluation despite being classified as no nodal evaluation (NNE)) were excluded, along with patients who had radiation or systemic therapies before or within 12 weeks after surgery. Our final cohort consisted of 4332 patients (Figure 1).

The management of the nodal basin was based on the type of surgery patients received (defined by the code RX_SUMM_SCOPE_REG_LN_2012 in NCDB) and classified as SLNB (codes 2, 6, and 7), ELND (codes 3, 4, and 5), or NNE (code 0). Per NCDB definition, the SLNB group was defined as patients who underwent “a procedure using injection of a dye, radio label, or combination to identify a lymph node (possibly more than one) for removal/examination.” These patients may have also had regional lymph nodes removed concurrently with the SLNB or after the SLNB. The group we defined as ELND was described as patients who had a regional lymph node dissection procedure without a SLNB (no blue dye or radio label used) concurrently or prior to the removal of regional lymph nodes. The NNE group was defined as patients who received no regional lymph node surgery. When patients were found to have nodal metastases after a SLNB, considered a + SLN, some underwent a regional lymph node dissection: CLND or no additional surgery of their nodal basin: observation.

Moreover, the baseline health status of patients was assessed by analyzing the average age of patients in each group and through the Charlson‐Deyo Comorbidity Scale, which incorporates 17 different comorbidities (not including cancer) to provide each patient with a numerical score summarizing the impact of their specific comorbidities. Clinical excision margin size was determined from the surgical procedure of the primary site by the surgeon (defined by the code RX_SUMM_SURG_PRIM_SITE in the NCDB) and classified as follows: less than or equal to 1.0 cm (codes 20, 27, 30, 31, 32, and 33), larger than 1.0 cm (code 45), 1.1–2.0 cm (code 46), and larger than 2.0 cm (code 47). This study was deemed exempt by the guidelines set forth by our Institutional Review Board.

### 2.2 Statistical Analysis

Baseline characteristics were compared between groups using one‐way ANOVA or chi‐squared test using Monte Carlo simulation for continuous and categorical variables, respectively. Multivariable logistic regression analyses were performed to examine factors that contribute to performance and type of lymph node evaluation, presence of positive lymph nodes, and strategy for management of a + SLN.

The follow‐up time was calculated as the duration from surgery to death, with censoring of patients without a record of death at the time of last known follow‐up. OS was estimated using the Kaplan–Meier (KM) method and compared between groups using the log‐rank test. Associations of nodal basin management strategy, tumor thickness, and surgical procedures with OS were examined using univariable, followed by multivariable Cox proportional‐hazards regression, with adjustment for patient and treatment characteristics associated with survival in univariable analysis. We also compared the OS of patients with a + SLN who underwent a CLND to those with no additional surgery and evaluated factors associated with a + SLN.

All tests were two‐sided, and statistical significance was set at *p* < 0.05. Multivariable regressions retained the main grouping variables as well as any other factors that had marginally significant (*p* < 0.1) associations with the outcome in univariable analysis. All statistical analyses were performed using R software, version 4.2.2.

## 3 Results

### 3.1 Patient Characteristics

Of the 4332 patients, most were self‐defined as White (4219; 97.4%), male (2880; 66.5%), ≥ 60‐year‐old (2734; 63.1%) and had no comorbidities (Charlson‐Deyo Score = 0) (3398; 78.4%). The median Breslow thickness of the primary melanoma of the entire cohort was 5.7 mm and 26% (1127) of patients had Breslow depth greater than 8 mm. The majority of patients had ulcerated primary tumors (2541; 58.7%) that were most often localized on extremities (1726; 39.8%), followed by trunk (1530; 35.3%) and the head and neck area (1047; 24.2%). The most common histologic subtype in patients was nodular melanoma (1771; 40.8%) followed by superficial spreading (578; 13.3%), desmoplastic (287; 6.6%), acral (67; 1.5%), and lentigo (52; 1.2%) melanomas. The median follow‐up time of the entire cohort was 33.2 months (IQR 21.0–50.2), and 34.8 (IQR 22.7–51.4), 32.6 (IQR 19.6–49.3) and 27.8 (IQR 15.1–45.0) months for patients in the SLNB, ELND and NNE group, respectively. Additionally, the median OS of all patients was 83.8 months.

Clinical characteristics and pathological features are summarized in Table 1.

**TABLE 1:** Characteristics of study patients stratified by no nodal evaluation (NNE), sentinel lymph node biopsy (SLNB), or elective lymph node dissection (ELND).

	All (*n* = 4332)	NNE (*n* = 682)	SLNB (*n* = 2851)	ELND (*n* = 799)	*p*
Age (%)					
18–59	1598 (36.89%)	159 (23.31%)	1098 (38.51%)	341 (42.68%)	< 0.001
60–69	1304 (30.1%)	192 (28.15%)	862 (30.24%)	250 (31.29%)	
70–80	1430 (33.01%)	331 (48.53%)	891 (31.25%)	208 (26.03%)	
Gender (%)					
Male	2880 (66.48%)	443 (64.96%)	1898 (66.57%)	539 (67.46%)	0.578
Female	1452 (33.52%)	239 (35.04%)	953 (33.43%)	260 (32.54%)	
Breslow thickness in mm (%)					
[4, 5)	1308 (30.19%)	163 (23.9%)	903 (31.67%)	242 (30.29%)	< 0.001
[5, 6)	962 (22.21%)	131 (19.21%)	651 (22.83%)	180 (22.53%)	
[6, 7)	546 (12.6%)	86 (12.61%)	367 (12.87%)	93 (11.64%)	
[7, 8)	389 (8.98%)	70 (10.26%)	245 (8.59%)	74 (9.26%)	
[8, 9)	290 (6.69%)	52 (7.62%)	185 (6.49%)	53 (6.63%)	
[9, Inf)	837 (19.32%)	180 (26.39%)	500 (17.54%)	157 (19.65%)	
Ulceration status (%)					
No	1742 (40.21%)	277 (40.62%)	1159 (40.65%)	306 (38.3%)	0.482
Yes	2541 (58.66%)	395 (57.92%)	1663 (58.33%)	483 (60.45%)	
Unknown/NA^a^	49 (1.13%)	10 (1.47%)	29 (1.02%)	10 (1.25%)	
Primary site (%)					
Trunk	1530 (35.32%)	202 (29.62%)	1030 (36.13%)	298 (37.3%)	< 0.001
Upper/lower extremities	1726 (39.84%)	222 (32.55%)	1206 (42.3%)	298 (37.3%)	
Other/NOS	29 (0.67%)	12 (1.76%)	13 (0.46%)	4 (0.5%)	
Head and neck	1047 (24.17%)	246 (36.07%)	602 (21.12%)	199 (24.91%)	
LVI (%)					
No	3084 (71.19%)	473 (69.35%)	2083 (73.06%)	528 (66.08%)	< 0.001
Yes	577 (13.32%)	93 (13.64%)	348 (12.21%)	136 (17.02%)	
Unknown^b^	671 (15.49%)	116 (17.01%)	420 (14.73%)	135 (16.9%)	
Histology group (%)					
Malignant melanoma NOS	1577 (36.4%)	255 (37.39%)	1004 (35.22%)	318 (39.8%)	< 0.001
Nodular	1771 (40.88%)	279 (40.91%)	1184 (41.53%)	308 (38.55%)	
Lentigo	52 (1.2%)	14 (2.05%)	25 (0.88%)	13 (1.63%)	
Superficial spreading	578 (13.34%)	66 (9.68%)	404 (14.17%)	108 (13.52%)	
Acral	67 (1.55%)	6 (0.88%)	45 (1.58%)	16 (2%)	
Desmoplastic NOS	287 (6.63%)	62 (9.09%)	189 (6.63%)	36 (4.51%)	
Charlson‐Deyo score (%)					
0	3398 (78.44%)	497 (72.87%)	2266 (79.48%)	635 (79.47%)	< 0.001
1	681 (15.72%)	113 (16.57%)	441 (15.47%)	127 (15.89%)	
2+	253 (5.84%)	72 (10.56%)	144 (5.05%)	37 (4.63%)	

^a^ Unknown/NA: Information not collected/not documented in patient record.

^b^ Unknown: LVI was not determined/documented in the patient record.

### 3.2 Wide Excision Surgery

**FIGURE 2:**
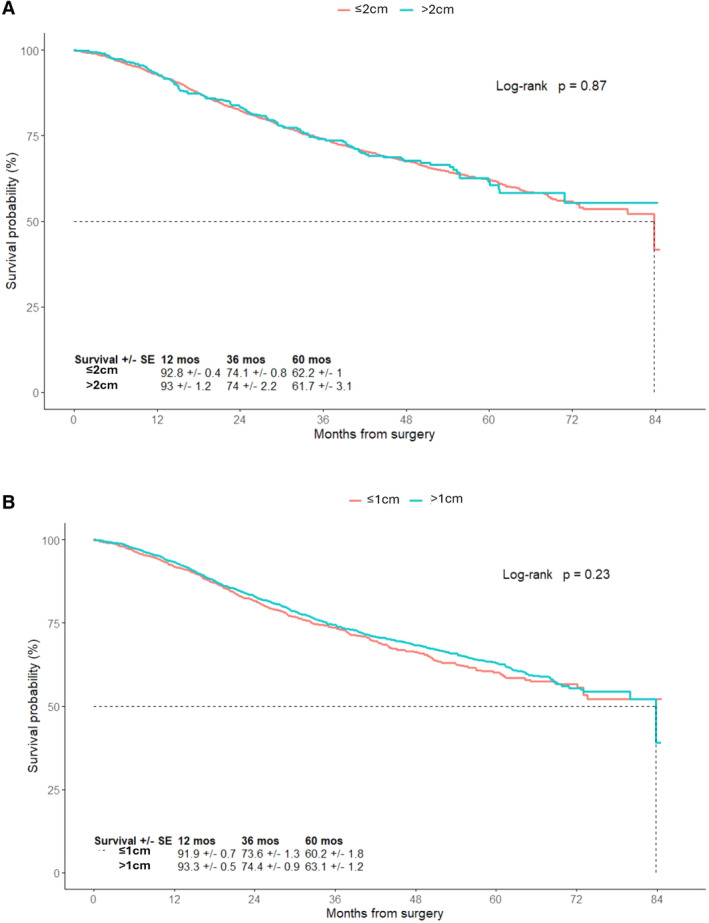
Overall survival (OS) according to size of the excision margins of the primary tumor. (A) Compares the OS of patients with excision margins ≤ 2 cm or > 2 cm. (B) Compares the OS of patients with excision margins ≤ 1 cm or > 1 cm.

The NCDB provided information regarding the size of excision margins that are based on the surgeon's estimation. The majority of patients had recorded excision margins less than 2 cm (89%) and a third had excision margins less than 1 cm (33.2%). Patients were categorized based on the size of the surgical margins: > 1 or ≤ 1 cm, or > 2 or ≤ 2 cm. Patients who had excision margins > 2 cm or ≤ 2 cm had similar 5‐year OS (61.7% ± 3.1% vs. 62.2% ± 1%; *p* = 0.87). The 5‐year OS of patients who had excision margins > 1 cm or ≤ 1 cm were 63.1% ± 1.2% versus 60.2% ± 1.8%, respectively (*p* = 0.23). There was no significant difference in OS based on the excision margin size of the primary tumor (Figure 2A and 2B).

A greater proportion of patients in the NNE group had positive surgical margins after wide excision (8.8%) compared to patients in the ELND (3.8%) and the SLNB (1.7%) groups (*p* < 0.001). However, positive surgical margins did not significantly impact OS on multivariable analysis.

### 3.3 Surgical Management of the Nodal Basin

The majority of patients underwent a SLNB (2851; 65.8%); the remainder had either an ELND (799; 18.4%) or NNE (682; 15.7%) (Figure 1). NNE patients were generally older with an average age of 66 [24–80] compared to 61 [18–80] and 60 [19–80] in the SLNB and ELND groups, respectively (*p* < 0.001). NNE patients were more likely to have multiple comorbidities (Charlson‐Deyo 2+: 10.6% vs. 5.1% and 4.6% for SLNB and ELND, respectively; *p* < 0.001) and to have melanoma in the head and neck region compared with patients who had a SLNB or an ELND (36.1% vs. 21.1% and 24.9% for SLNB and ELND, respectively; *p* < 0.001) (Table 1).

**FIGURE 3:**
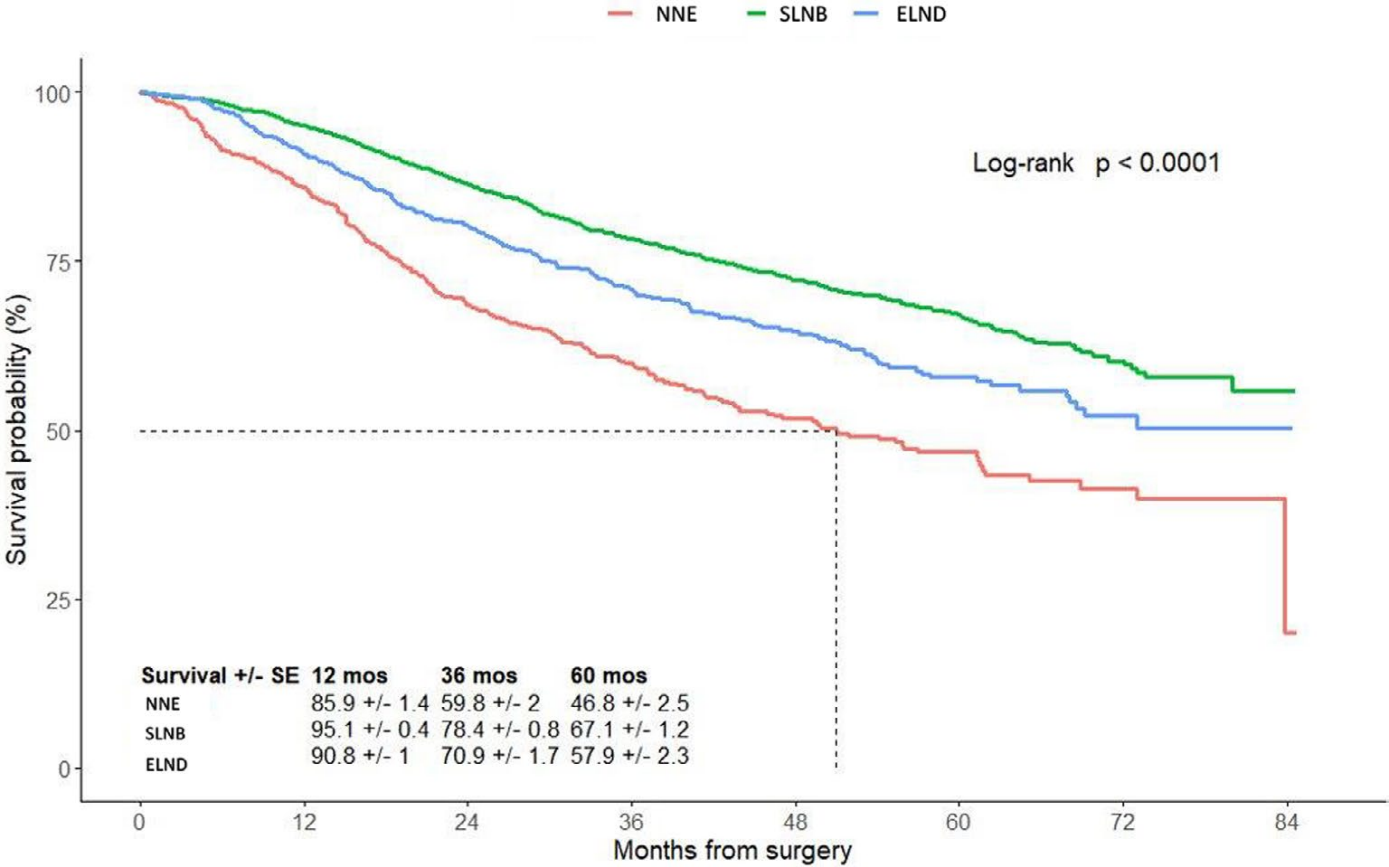
Overall survival according to surgical management of the lymph node basin. Comparison of patients who had sentinel lymph node biopsy (SLNB, *n* = 2851), an elective lymph node dissection (ELND, *n* = 799), and patients who did not have any surgical nodal evaluation (NNE, *n* = 682).

The 5‐year estimated OS for patients who had a SLNB (67.1% ± 1.2%) or an ELND (57.9% ± 2.3%) was better compared to patients with NNE (46.8% ± 2.5%; *p* < 0.001) (Figure 3). Similar results were observed when we examined the 5‐year OS among the different surgical treatment per age group. As show in Figure S1, for patients between 18 and 59 years old, those who had a SLNB had a better 5‐year OS (76.3% ± 1.7%) compared to ELND (61.8% ± 3.6%) and NNE (62.9% ± 4.7%) (*p* < 0.0001). Similar trends were noted for patients between 60 and 69 years old and patients between 70 and 80 years old (Figure S1). Additionally, we showed the same trends for patients with and without comorbidities (Figure S2).

**FIGURE 4:**
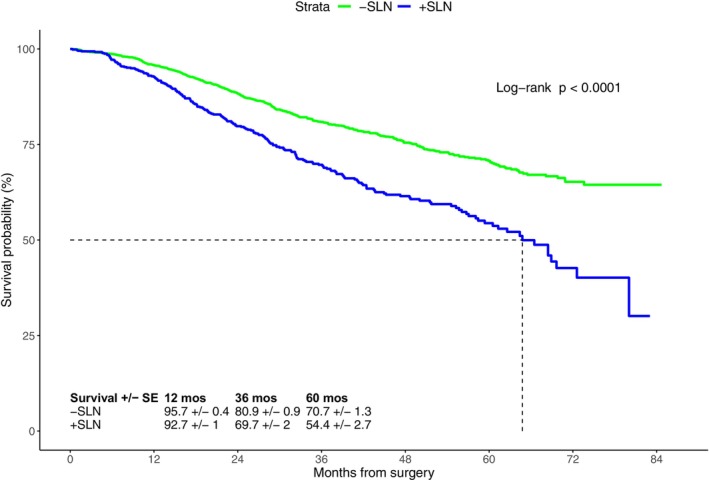
Overall survival according to status of the sentinel nodes: 2156 patients had no metastases in their lymph nodes after SLNB (−SLN group, in green), the 643 patients had metastases in their lymph nodes after SLNB (+ SLN, in blue).

As expected, patients with a tumor‐negative SLN (−SLN) had better 5‐year survival (70.7% ± 1.3%) compared to patients with a + SLN (54.4% ± 2.7%; Figure 4). Similarly, patients in the ELND group with tumor‐negative regional nodes had better 5‐year OS compared to patients with tumor‐positive regional nodes (66.5% ± 3.1% vs. 49.2% ± 3.6%; *p* < 0.001) The 5‐year OS of patients with primary tumor thicknesses of 4.0–4.9 mm, 5–5.9 mm, 6–6.9 mm, 7–7.9 mm, 8–8.9 mm, and ≥ 9 mm were 68.2% ± 1.8%, 62.9% ± 2.1%, 61.5% ± 2.7%, 57.5% ± 3.3%, 60% ± 3.7%, and 55.3% ± 2.3%, respectively; showing that with increasing thickness, the 5‐year OS significantly decreased (*p* < 0.0001, Figure S3).

Multivariable analysis revealed that ulcerated primary tumors (aHR 1.59, 95% CI 1.17–2.17), lymphovascular invasion (LVI) (aHR 1.4, 95% CI 1.2–1.63), increasing Breslow thickness ≥ 6 mm (6–6.9 mm: aHR 1.24, 95% CI 1.02–1.51; 7–7.9 mm: aHR 1.46, 95% CI 1.18–1.79; 8–8.9 mm: aHR 1.54, 95% CI 1.22–1.95; ≥ 9 mm: aHR 1.56, 95% CI 1.32–1.84), nodal metastases (aHR 1.79, 95% CI 1.55–2.06), and older age (≥ 70 relative to 18–59; aHR 1.66, 95% CI 1.39–1.98) were associated with worse OS (Table S1). Meanwhile, factors associated with improved OS included primary tumor location on the extremity (compared to the trunk) (aHR 0.77, 95% CI 0.67–0.88), female gender (aHR 0.86, 95% CI 0.76–0.98), SLNB (compared to NNE group) (aHR 0.59, 95% CI 0.36–0.97), and desmoplastic histology (compared to malignant melanoma NOS) (aHR 0.61, 95% CI 0.44–0.84) (Table S1). The size of clinical margins used during wide excision surgery was not significantly associated with OS.

### 3.4 Management of Tumor‐Positive Sentinel Lymph Node

23% (643) of the patients who underwent a SLNB had a + SLN, and of those, 62.3% (400) of them had a CLND afterwards (Figure 1). The median Breslow thickness of patients with positive and negative SLNs was 5.5 mm for both groups. The +SLN rate of patients in different primary tumor thickness groups ranged from 22.57% to 23.33%. Thickness was not significantly associated with the status of the SLN on multivariable analysis (Table 2). Desmoplastic melanoma patients had a much lower rate of +SLN (4.28%), while patients with superficial spreading and acral melanomas had a much higher +SLN rate (33.16%; 38.64%).

**TABLE 2:** Variables associated with a tumor‐positive sentinel lymph node (+SLN) in patients with thick melanoma.

Variable	Univariable	*p*	Multivariable	*p*
	HR		HR	
Age				
18–59	Reference		Reference	
60–69	0.7	0.001	0.79	0.055
70–80	0.55	< 0.001	0.65	0.004
Thickness (mm)				
[4, 5)	Reference		Reference	
[5, 6)	1.01	0.912	1.04	0.774
[6, 7)	1.13	0.401	1.12	0.448
[7, 8)	0.92	0.641	0.95	0.777
[8, 9)	1.04	0.824	1.13	0.56
[9, Inf)	1.04	0.75	1.06	0.707
Ulceration				
No	Reference		Reference	
Yes	1.48	< 0.001	1.29	0.389
Primary site				
Trunk	Reference		Reference	
Extremities	0.83	0.051	0.85	0.12
Head/neck	0.42	< 0.001	0.51	< 0.001
Histology group				
Malignant melanoma NOS	Reference		Reference	
Nodular	1.06	0.595	0.95	0.653
Lentigo	1.13	0.8	1.36	0.529
Superficial spreading	1.77	< 0.001	1.47	0.006
Acral	2.25	0.011	1.78	0.92
Desmoplastic NOS	0.16	< 0.001	0.23	< 0.001
Lymphovascular invasion				
No	Reference		Reference	
Yes	2.69	< 0.001	2.38	< 0.001

On multivariable analysis, primary tumors localized on the head and neck (aOR 0.51, 95% CI 0.38–0.68, relative to trunk), desmoplastic histology (aOR 0.23, 95% CI 0.1–0.45, relative to malignant melanoma NOS) and older age (≥ 70 relative to 18–59; aOR 0.65, 95% CI 0.48–0.87) were associated with a −SLN. Only LVI (aOR 2.49, 95% CI 1.93–3.2) and superficial spreading histology subtype (aOR 1.47, 95% CI 1.11–1.94, relative to malignant melanoma NOS) were associated with a + SLN on multivariable analysis (Table 2).

The 5‐year OS of the CLND and observation groups were 53.3% ± 3.3% and 56.6% ± 4.4%, respectively (*p* = 0.67). Thus, there was no survival advantage associated with performance of a CLND in thick melanoma patients with a + SLN (Figure S3).

## 4 Discussion

Representing less than 5% of cutaneous melanoma cases [2], patients with thick primary tumors (> 4 mm, T4) have been underrepresented in most prospective surgical trials. The surgical management of these patients remains controversial, since surgery alone may not improve their survival [16, 17, 18, 19]. To our knowledge, this retrospective study using NCDB data from 2012 to 2017, is one of the largest studies investigating surgical options for thick melanoma patients. However, compared to other studies, such as Song et al., our study utilizes the code RX_SUMM_SCOPE_REG_LN_2012 to specify the type of nodal surgery patients received, addressing some limitations from previous analyses [15].

NCCN guidelines lack specificity regarding surgical excision margin size for thick melanoma patients, as T2b‐T4 melanomas are grouped together [20]. Currently, there is an ongoing international randomized clinical trial assessing surgical margins for thicker (≥ 2 mm) melanoma [21]. After evaluating various excision margin sizes, including ≤ 1 cm versus 1 > cm and ≤ 2 cm versus > cm, our results show no significant difference in five‐year OS based on surgical margin size. We look forward to the results of MelMarT trial, and perhaps further investigation should look at anatomical site (head/neck vs. trunk vs. extremities) to determine margins based on the estimated risk of local recurrence.

As expected, the majority of patients underwent a SLNB. These patients had a better 5‐year OS (67.1% ± 1.2%) compared to patients who had either an ELND (57.9% ± 2.3%) or NNE (46.8% ± 2.5%) (*p* < 0.0001). As previously described in MSLT‐I and several other studies, we confirmed the improved staging associated with a SLNB for patients with thick primary tumors [11, 16, 18, 19, 22, 23, 24, 25, 26, 27]. We showed similar results: a better 5‐year OS for patients who underwent SLNB compared to ELND or NNE, for each of the three age groups; confirming the importance of SLNB for all patients independently of their age. Our results agree with previous findings showing correlation between status of the SLN and OS in patients with primary tumors thicker than 4 mm [17, 23, 27]. Han et al., showed that SLN metastasis was always associated with worse outcome in all subgroups of thick melanoma [17, 23, 27]. A recent retrospective study done by Holmberg et al., with 1943 thick melanoma patients also suggest that SLN status in thick melanoma patients is predictive of survival [28]. Surprisingly, 18.4% of the patients in our dataset underwent ELND. Prior to the introduction of SLNB in 1992, ELND was the standard of care for cutaneous melanoma. However, since the development of SLNB and MSLT‐I clinical trial, ELND has been replaced by SLNB for patients with clinically node‐negative disease [20, 29]. Because of the time period of our study, it is unclear why any patients had an ELND. Unfortunately, the NCDB does not include the reason why surgeons decided on the type of nodal assessment performed. Hopefully, the trend will be towards less frequent use of ELND in the future.

As expected, we found that patients who had a + SLN had a significantly decreased OS compared to patients with a −SLN (Figure 4). Surprisingly, the +SLN group had a better OS compared to the NNE group. The ELND group showed a similar trend, with patients with tumor‐positive regional nodes having poorer OS than those with tumor‐negative regional nodes.

In our study, 23% of SLNB patients had nodal metastases (Figure 1). This positivity rate differs from the findings of Gyorki et al., who conducted a meta‐analysis including 10 studies and found that the +SLN rate ranged from 33%–52% [30]. We speculate this may be due to our cohort having older patients, with more primary tumors on the head/neck region. Factors associated with +SLNs included ulceration and LVI, while location on the head/neck and extremities along with older age (≥ 60) were associated with −SLNs (Table 2). These findings are consistent with those of previous studies [16, 19, 22, 24, 31, 32, 33, 34, 35].

Of these 643 patients with a + SLN, 400 (62.3%) underwent a CLND, which did not appear to confer a significant OS benefit compared to the observation group (Figure S4). Our data correlate with the MSLT‐II [13] and DeCOG‐SLT [12] results, showing that a CLND is not associated with a survival benefit in patients with thick melanoma.

We observed a significant inverse association between Breslow thickness of the primary tumor and OS. Although other studies have examined the survival of thick melanoma patients in different thickness groups [22, 23, 26], our study is the first to show that each millimeter of additional thickness is adversely associated with the patients' OS (Figure S3). In addition to thickness, the multivariable analysis revealed that site and ulceration of the primary tumor, LVI, status of the SLN, gender, histology subtype, and age all significantly impact OS (Table S1).

There are several limitations to this analysis. First, as a retrospective NCDB study, many patient profiles lacked complete data. For instance, all primary tumors thicker than 9.8 mm were recorded and grouped as 9.8 mm, preventing specific analysis of how a tumor thicker than 9.8 mm affected OS. Second, because the cause of death was not recorded, we could only evaluate OS, rather than the melanoma specific survival or the times to local and/or distant recurrence, which creates a potential bias as some patients are older, and have shorter life expectancy. Furthermore, the rationale for treatment was based on the individual treating physician's decision that is not specified in NCDB. Therefore, it is possible that patients who had a significantly lower OS in the NNE group were deemed ineligible for a SLNB or ELND based on their health status. This may have influenced OS, as death may have resulted from factors other than melanoma.

Our results confirmed the staging and prognosis value of SLNB, and as recommended by the NCCN, SLNB should be discussed with all thick melanoma patients for whom the probability of nodal positivity is greater than 10%. These results are consistent with many studies describing the status of sentinel lymph nodes as the most significant prognostic tool for cutaneous melanoma [9, 11, 36, 37]. In the past decade, nomograms have been developed to try to predict SLN status [37, 38, 39, 40, 41, 42], but none are completely accurate (AUC varies from 0.68 to 0.87). El Sharouni et al., showed that knowing the status of the SLN improves the predictive accuracy of clinicopathological features alone. Dixon et al., suggested the lack of prognostic benefit of SLNB for young (< 40 years old) and old (> 60 years old) patients [38, 43]. Thus, the benefit of SLNB remains controversial. Risks associated with performing SLNBs should be considered before performing the procedure; even if they affect only a small percentage (around 11%) of patients and tend to be minor and short‐lived such as wound infection, seroma, some side effects can be long lasting such as cutaneous nerve injury, and lymphedema. In spite of these concerns, we believe that the staging benefits of SLNB, which have been reported to play a crucial role in guiding care by facilitating early identification of metastases and the use of systemic therapies [44, 45, 46] should be recommended or at least discussed with all thick melanoma patients [47, 48].

## 5 Conclusions

Among thick melanoma patients with clinically node‐negative, nonmetastatic, extensive discrepancies remain regarding the surgical management of the nodal basin. Increasing depth of invasion, presence of ulceration, primary tumor location, and nodal metastases were all found to be associated with worse OS, providing key pathological information. SLNB is a low morbidity procedure that should be discussed with all patients with a thick melanoma, as nodal status is prognostically important.

## Author Contributions

**Arthur W. Cowman:** conceptualization (equal), formal analysis (equal), methodology (equal), writing – original draft (lead), writing – review and editing (equal). **Kristel Lourdault:** conceptualization (equal), formal analysis (equal), methodology (equal), writing – original draft (supporting), writing – review and editing (equal). **Douglas Hanes:** data curation (equal), formal analysis (equal), methodology (equal), writing – review and editing (equal). **Jessica Weiss:** conceptualization (equal), formal analysis (equal), methodology (equal), writing – original draft (supporting), writing – review and editing (supporting). **Sean Nassoiy:** conceptualization (equal), data curation (lead), formal analysis (supporting), methodology (supporting), writing – review and editing (supporting). **Melanie Goldfarb:** data curation (supporting), writing – review and editing (supporting). **Richard Essner:** conceptualization (lead), methodology (lead), supervision (equal), writing – review and editing (equal).

## Conflicts of Interest

RE serves on the advisory board for Castle Biosciences and IntraMedical Imaging. The remaining authors have no conflicts of interest to report.

## Supporting information


**Figure S1.** Overall Survival according to surgical management of the lymph node basin per age group. Comparison of patients who had sentinel lymph node biopsy (SLNB), an elective lymph node dissection (ELND), and patients who did not have any surgical nodal evaluation (NNE) per age group; (A) patients between 18 and 59 years old, (B) patients between 60 and 69 years old and (C) patients between 70 and 70 years old.

**Figure S2.** Overall Survival according to surgical management of the lymph node basin per comorbidity score. Comparison of patients who had sentinel lymph node biopsy (SLNB), an elective lymph node dissection (ELND), and patients who did not have any surgical nodal evaluation (NNE) per comorbidity score; (A) patients with no comorbidity and (B) patients with at least one comorbidity.

**Figure S3.** Overall Survival according to the thickness of the primary tumor. Comparison of OS between thick melanoma patients with varying levels of tumor thickness.

**Figure S4.** Overall Survival of patients with a + SLN according to their treatment: CLND (in red) or observation (in blue).

**Table S1.** Variables associated with overall survival in thick melanoma patients.

## Data Availability

The National Cancer Database was queried from the years 2012 to 2017 to identify patients ages 18 to 80 with a diagnosis of thick primary cutaneous melanoma (cT4) who were clinically node‐negative (cN0) and without known metastatic (cM0) disease. This data is publicly available.
