# Supplementary figures and images for: Surgical Management of Thick Primary Cutaneous Melanoma in the US
Source: Cancer Med. 2025 Feb 20;14(4):e70578. doi: 10.1002/cam4.70578 (PMC11840694; doi:10.1002/cam4.70578)

A

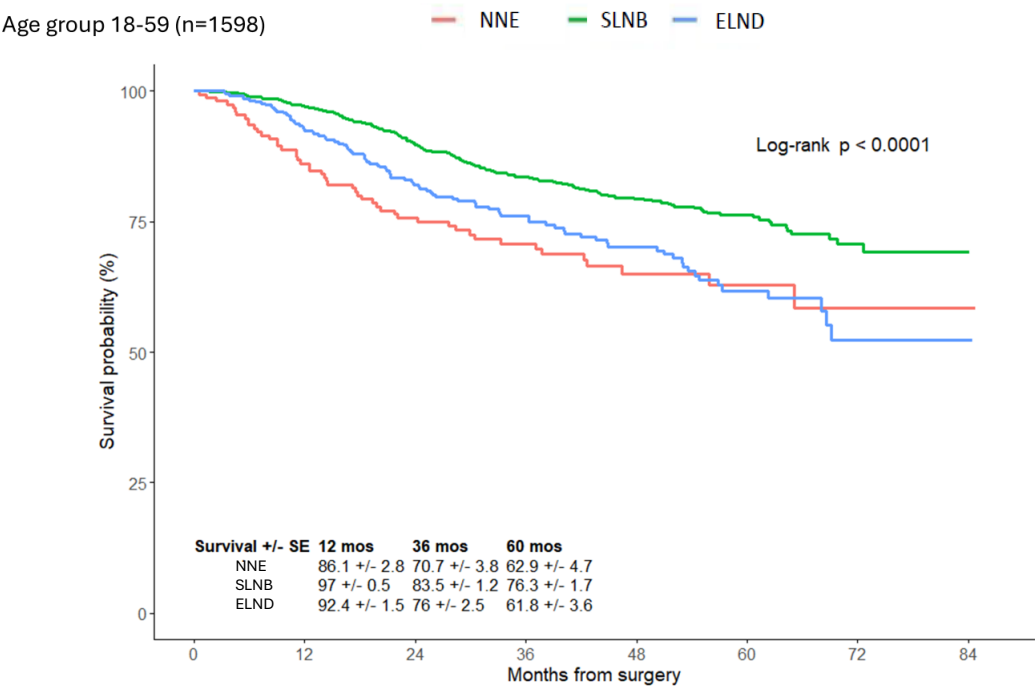

B

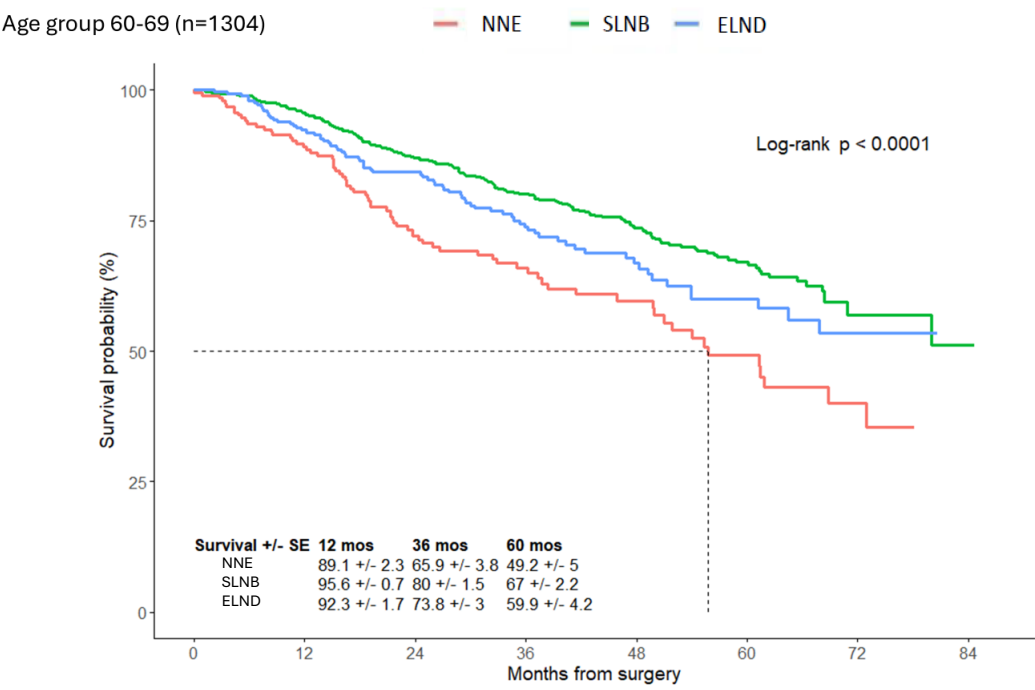

C

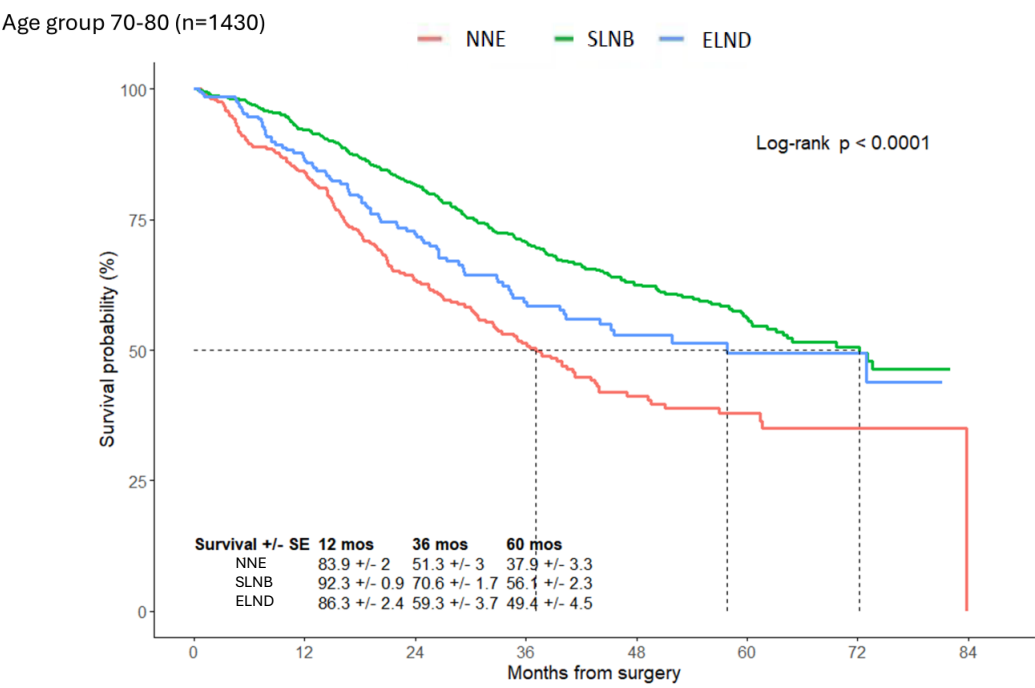

Supplement: Supplementary file 1 — Figure S1. Overall Survival according to surgical management of the lymph node basin per age group. Comparison of patients who had sentinel lymph node biopsy (SLNB), an elective lymph node dissection (ELND), and patients who did not have any surgical nodal evaluation (NNE) per age group; (A) patients between 18 and 59 years old, (B) patients between 60 and 69 years old and (C) patients between 70 and 70 years old. [file CAM4-14-e70578-s004.pdf]

**A**

No comorbidities (n=3398)

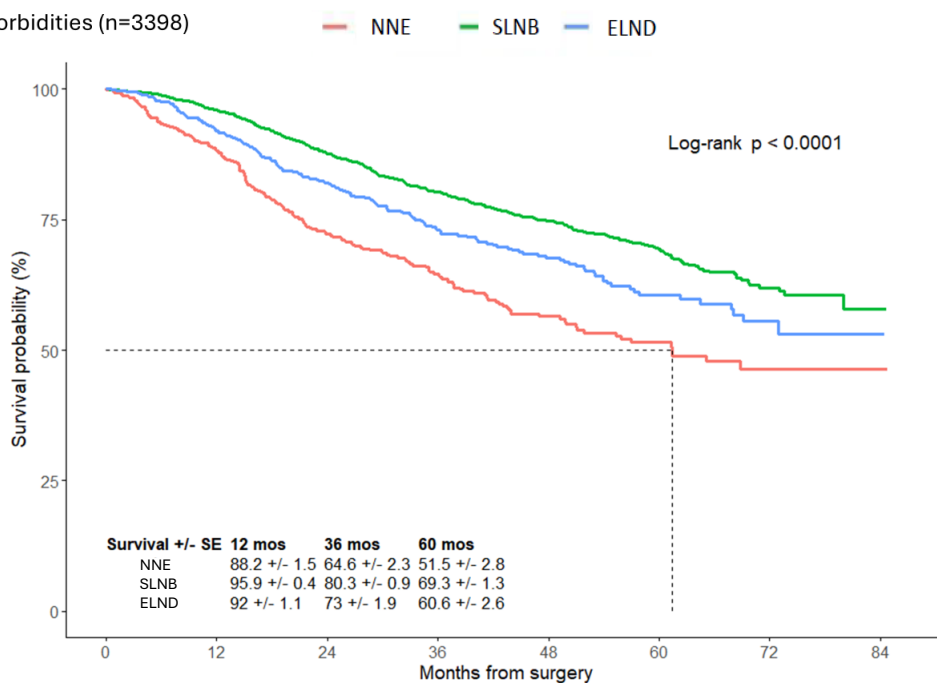**B**

At least one comorbidity (n=934)

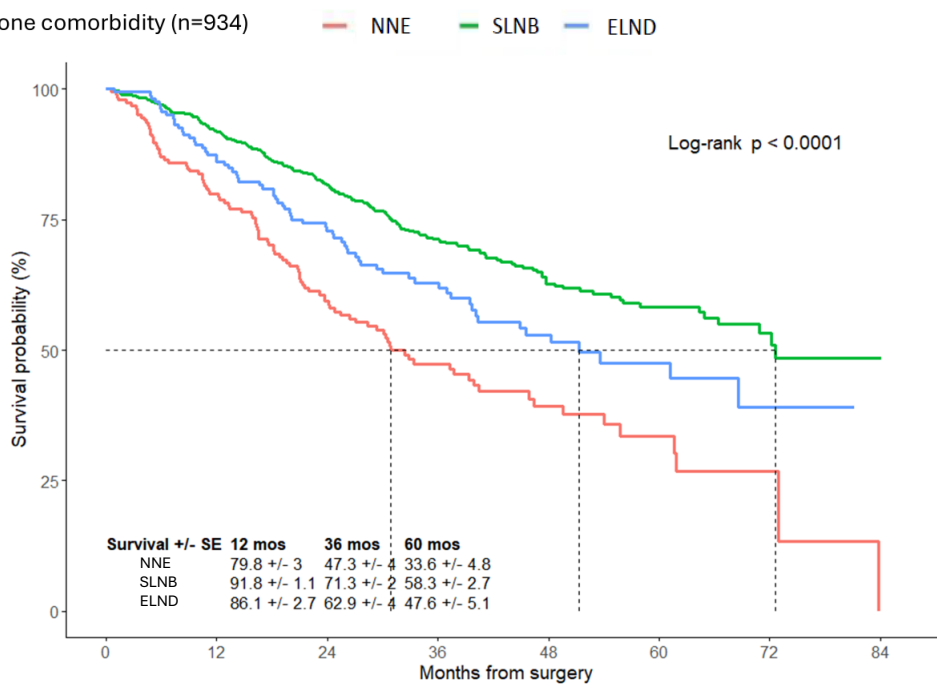

Supplement: Supplementary file 2 — Figure S2. Overall Survival according to surgical management of the lymph node basin per comorbidity score. Comparison of patients who had sentinel lymph node biopsy (SLNB), an elective lymph node dissection (ELND), and patients who did not have any surgical nodal evaluation (NNE) per comorbidity score; (A) patients with no comorbidity and (B) patients with at least one comorbidity. [file CAM4-14-e70578-s005.pdf]

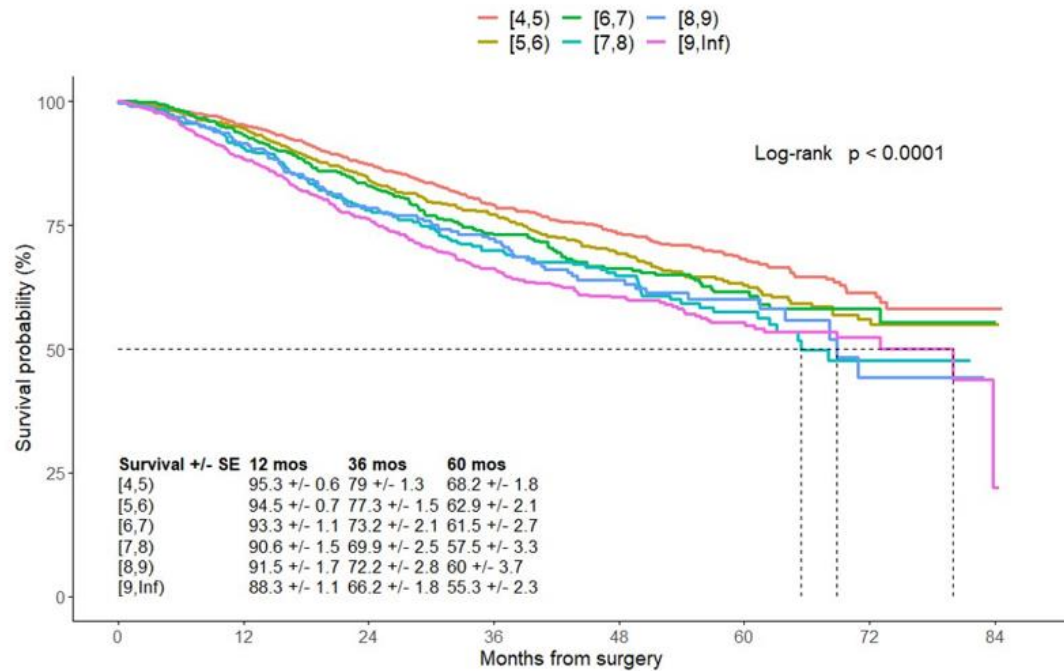

Supplement: Supplementary file 3 — Figure S3. Overall Survival according to the thickness of the primary tumor. Comparison of OS between thick melanoma patients with varying levels of tumor thickness. [file CAM4-14-e70578-s003.pdf]

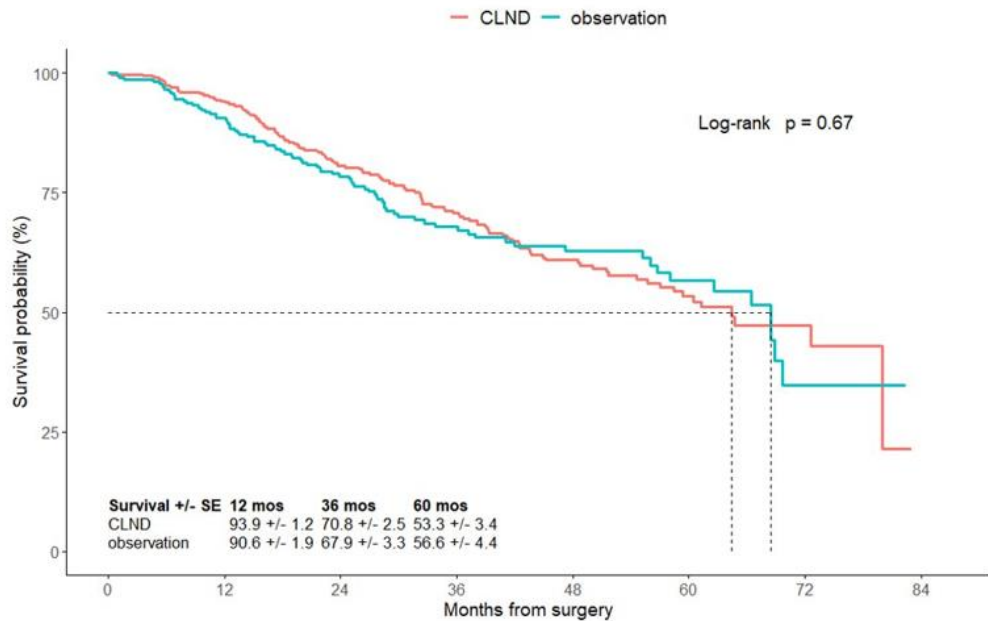

Supplement: Supplementary file 4 — Figure S4. Overall Survival of patients with a + SLN according to their treatment: CLND (in red) or observation (in blue). [file CAM4-14-e70578-s001.pdf]
