# Supplementary material for: Surgical Management of Thick Primary Cutaneous Melanoma in the US
Source: Cancer Med. 2025 Feb 20;14(4):e70578. doi: 10.1002/cam4.70578 (PMC11840694; doi:10.1002/cam4.70578)
Supplement: Supplementary file 5 — Table S1. Variables associated with overall survival in thick melanoma patients. [file CAM4-14-e70578-s002.docx]

**Supplemental Table 1. Variables associated with overall survival in thick melanoma patients.**
